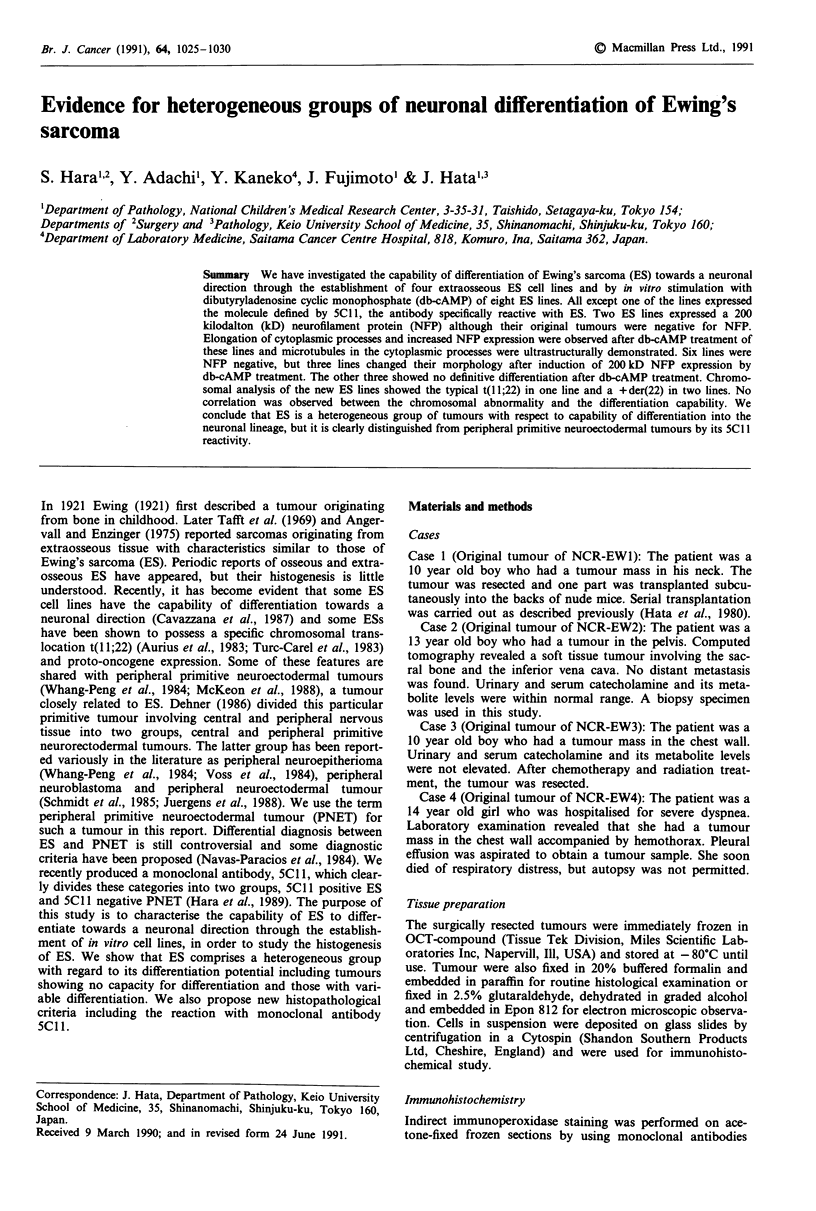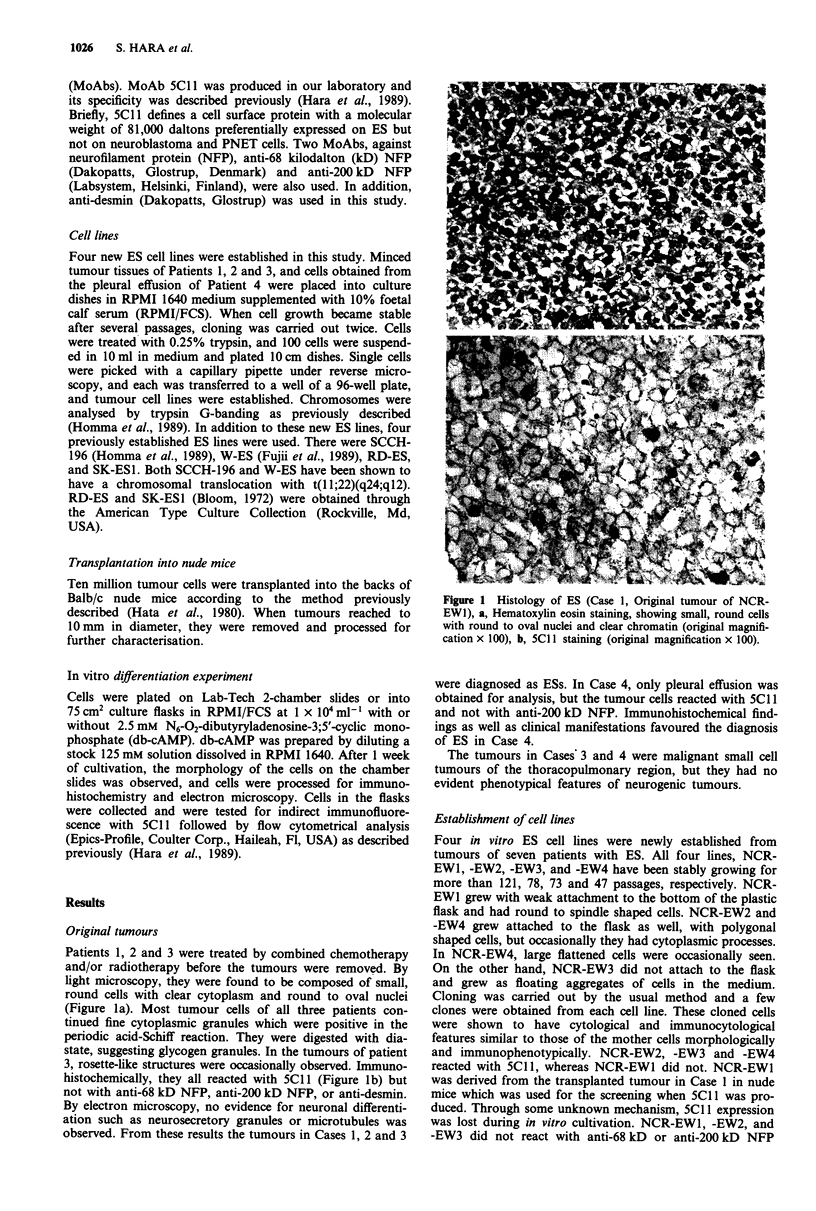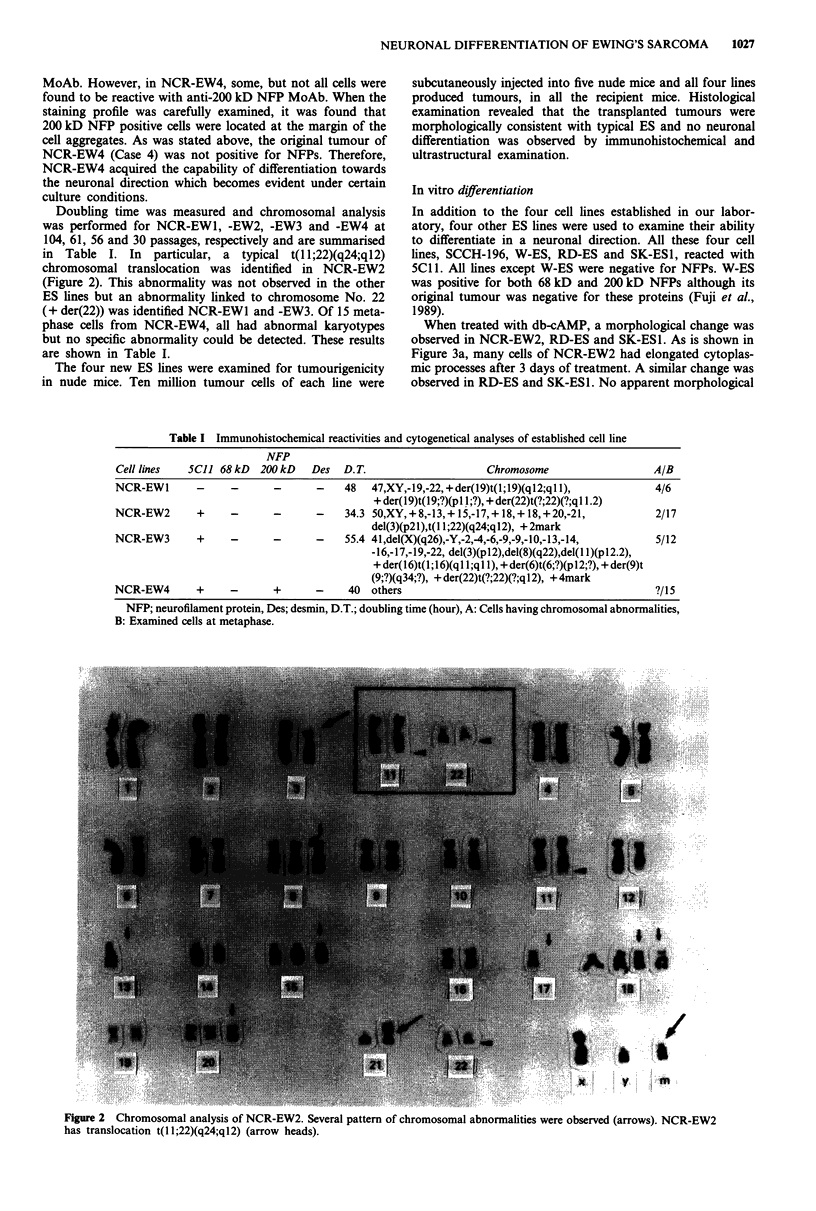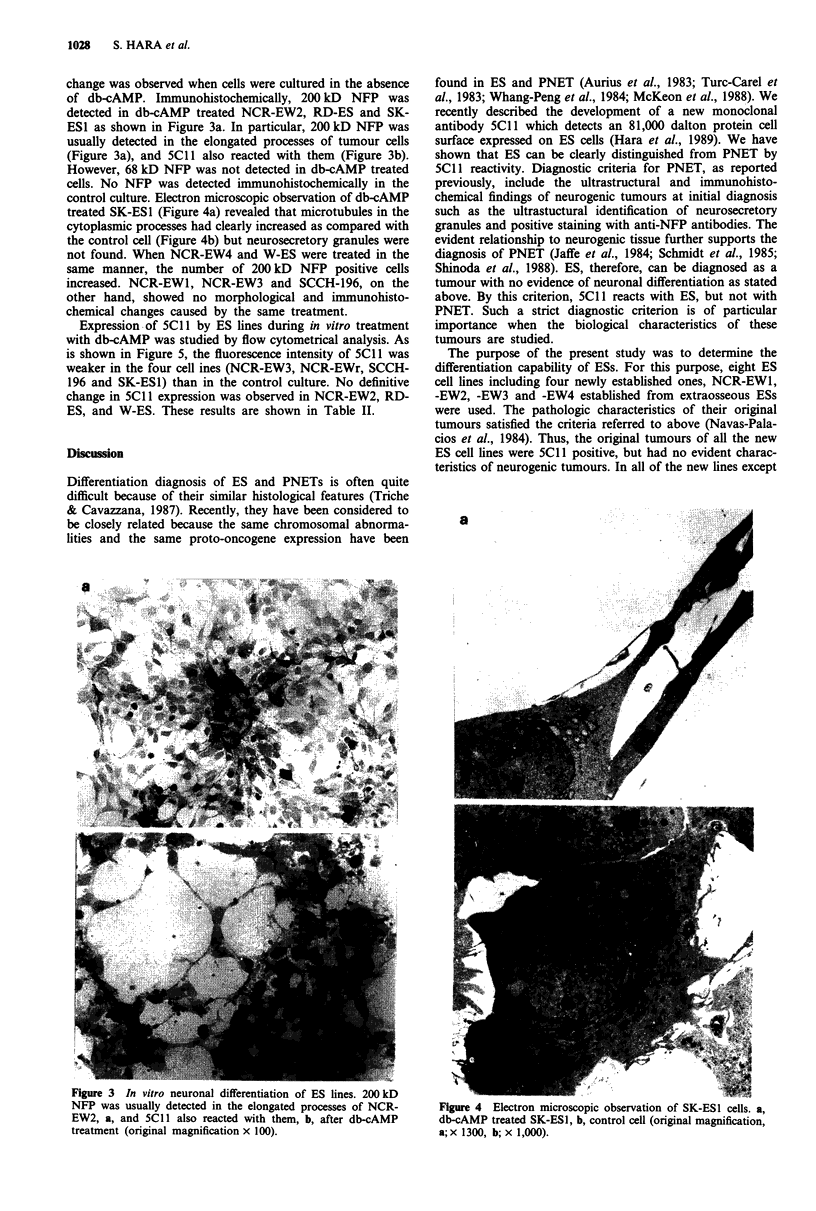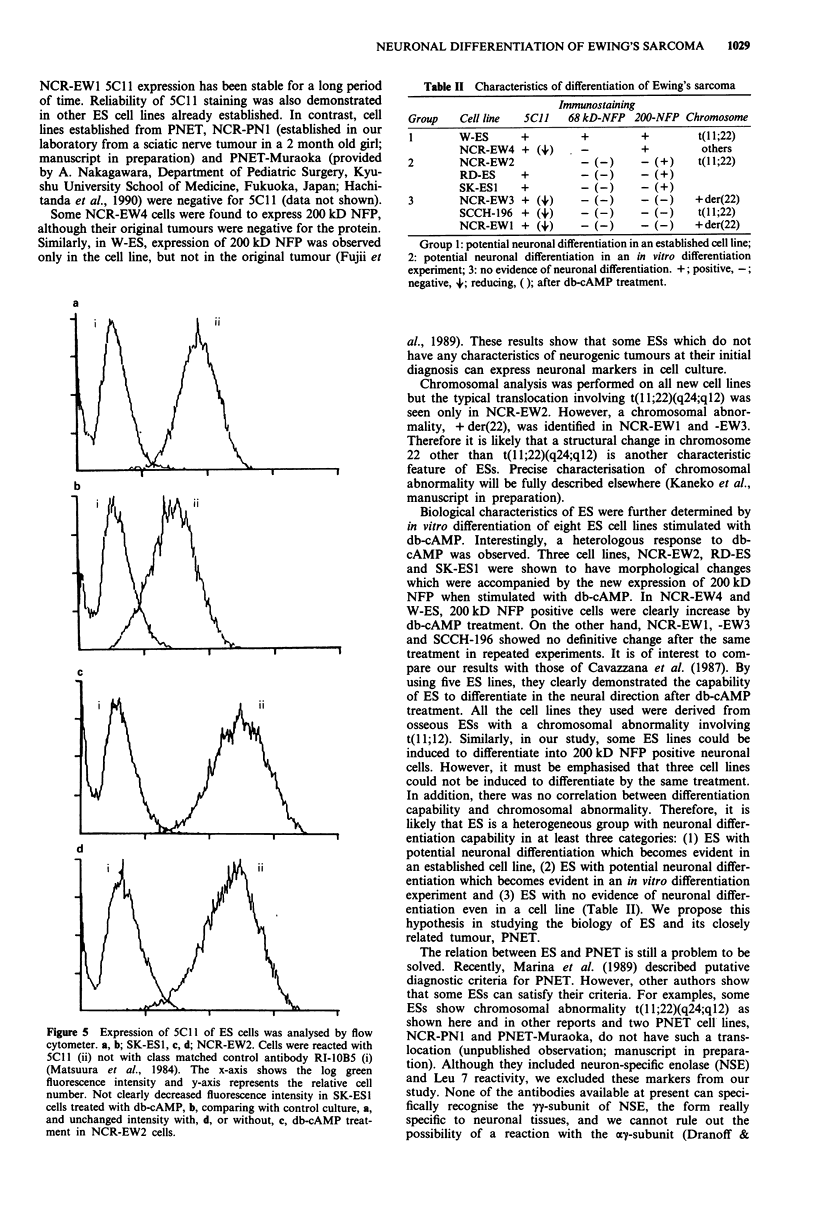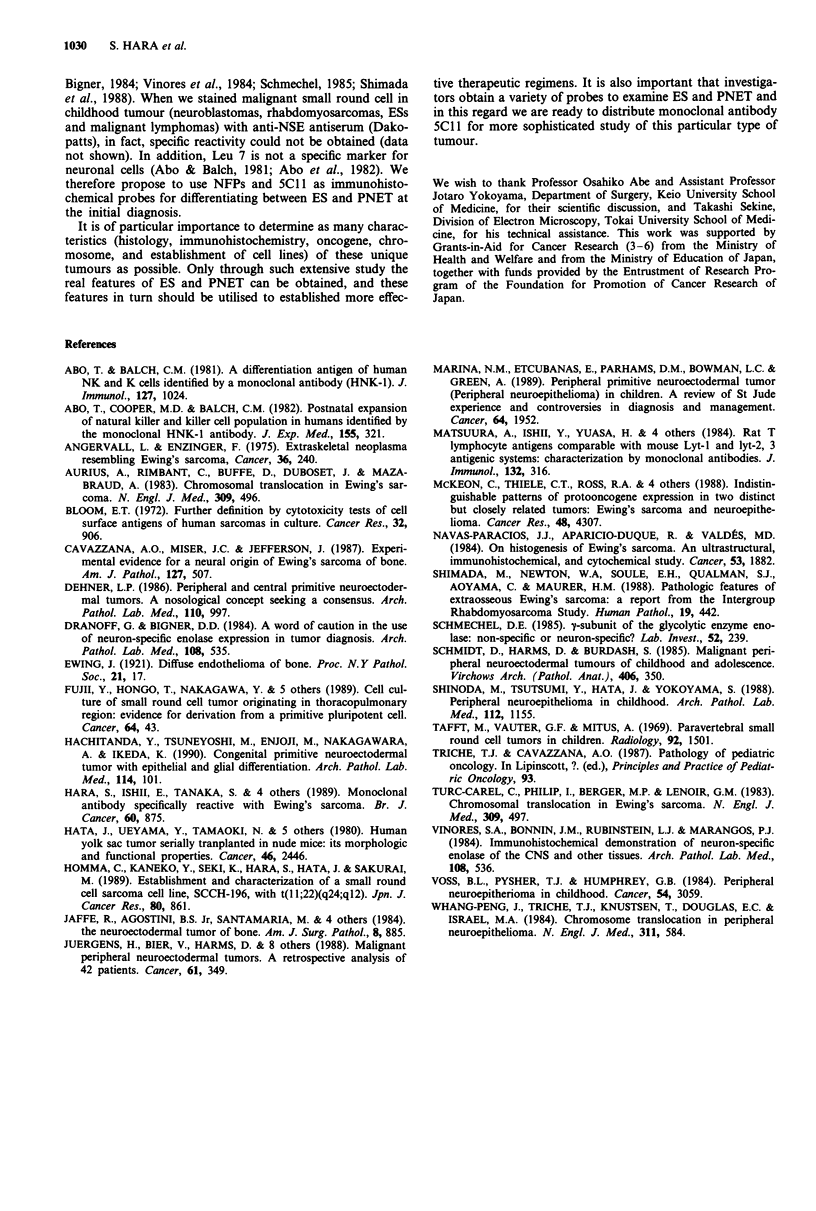# Evidence for heterogeneous groups of neuronal differentiation of Ewing's sarcoma.

**DOI:** 10.1038/bjc.1991.458

**Published:** 1991-12

**Authors:** S. Hara, Y. Adachi, Y. Kaneko, J. Fujimoto, J. Hata

**Affiliations:** Department of Pathology, National Children's Medical Research Center, Tokyo, Japan.

## Abstract

**Images:**


Br. J. Cancer (1991), 64, 1025-1030 © Macmillan Press Ltd., 1991

### Evidence for heterogeneous groups of neuronal differentiation of Ewing's sarcoma

S. Hara[1,2], Y. Adachi[1], Y. Kaneko[4], J. Fujimoto[1] & J. Hata[1,3]

[1]*Department of Pathology, National Children's Medical Research Center, 3-35-31, Taishido, Setagaya-ku, Tokyo 154; Departments of [2]Surgery and [3]Pathology, Keio University School of Medicine, 35, Shinanomachi, Shinjuku-ku, Tokyo 160; [4]Department of Laboratory Medicine, Saitama Cancer Centre Hospital, 818, Komuro, Ina, Saitama 362, Japan.*

**Summary** We have investigated the capability of differentiation of Ewing's sarcoma (ES) towards a neuronal direction through the establishment of four extraosseous ES cell lines and by *in vitro* stimulation with dibutyryladenosine cyclic monophosphate (db-cAMP) of eight ES lines. All except one of the lines expressed the molecule defined by 5C11, the antibody specifically reactive with ES. Two ES lines expressed a 200 kilodalton (kD) neurofilament protein (NFP) although their original tumours were negative for NFP. Elongation of cytoplasmic processes and increased NFP expression were observed after db-cAMP treatment of these lines and microtubules in the cytoplasmic processes were ultrastructurally demonstrated. Six lines were NFP negative, but three lines changed their morphology after induction of 200 kD NFP expression by db-cAMP treatment. The other three showed no definitive differentiation after db-cAMP treatment. Chromosomal analysis of the new ES lines showed the typical t(11;22) in one line and a +der(22) in two lines. No correlation was observed between the chromosomal abnormality and the differentiation capability. We conclude that ES is a heterogeneous group of tumours with respect to capability of differentiation into the neuronal lineage, but it is clearly distinguished from peripheral primitive neuroectodermal tumours by its 5C11 reactivity.

In 1921 Ewing (1921) first described a tumour originating from bone in childhood. Later Tafft *et al.* (1969) and Angervall and Enzinger (1975) reported sarcomas originating from extraosseous tissue with characteristics similar to those of Ewing's sarcoma (ES). Periodic reports of osseous and extraosseous ES have appeared, but their histogenesis is little understood. Recently, it has become evident that some ES cell lines have the capability of differentiation towards a neuronal direction (Cavazzana *et al.*, 1987) and some ESs have been shown to possess a specific chromosomal translocation t(11;22) (Aurius *et al.*, 1983; Turc-Carel *et al.*, 1983) and proto-oncogene expression. Some of these features are shared with peripheral primitive neuroectodermal tumours (Whang-Peng *et al.*, 1984; McKeon *et al.*, 1988), a tumour closely related to ES. Dehner (1986) divided this particular primitive tumour involving central and peripheral nervous tissue into two groups, central and peripheral primitive neurorectodermal tumours. The latter group has been reported variously in the literature as peripheral neuroepitherioma (Whang-Peng *et al.*, 1984; Voss *et al.*, 1984), peripheral neuroblastoma and peripheral neuroectodermal tumour (Schmidt *et al.*, 1985; Juergens *et al.*, 1988). We use the term peripheral primitive neuroectodermal tumour (PNET) for such a tumour in this report. Differential diagnosis between ES and PNET is still controversial and some diagnostic criteria have been proposed (Navas-Paracios *et al.*, 1984). We recently produced a monoclonal antibody, 5C11, which clearly divides these categories into two groups, 5C11 positive ES and 5C11 negative PNET (Hara *et al.*, 1989). The purpose of this study is to characterise the capability of ES to differentiate towards a neuronal direction through the establishment of *in vitro* cell lines, in order to study the histogenesis of ES. We show that ES comprises a heterogeneous group with regard to its differentiation potential including tumours showing no capacity for differentiation and those with variable differentiation. We also propose new histopathological criteria including the reaction with monoclonal antibody 5C11.

Correspondence: J. Hata, Department of Pathology, Keio University School of Medicine, 35, Shinanomachi, Shinjuku-ku, Tokyo 160, Japan.
Received 9 March 1990; and in revised form 24 June 1991.

### Materials and methods

### *Cases*

Case 1 (Original tumour of NCR-EW1): The patient was a 10 year old boy who had a tumour mass in his neck. The tumour was resected and one part was transplanted subcutaneously into the backs of nude mice. Serial transplantation was carried out as described previously (Hata *et al.*, 1980).

Case 2 (Original tumour of NCR-EW2): The patient was a 13 year old boy who had a tumour in the pelvis. Computed tomography revealed a soft tissue tumour involving the sacral bone and the inferior vena cava. No distant metastasis was found. Urinary and serum catecholamine and its metabolite levels were within normal range. A biopsy specimen was used in this study.

Case 3 (Original tumour of NCR-EW3): The patient was a 10 year old boy who had a tumour mass in the chest wall. Urinary and serum catecholamine and its metabolite levels were not elevated. After chemotherapy and radiation treatment, the tumour was resected.

Case 4 (Original tumour of NCR-EW4): The patient was a 14 year old girl who was hospitalised for severe dyspnea. Laboratory examination revealed that she had a tumour mass in the chest wall accompanied by hemothorax. Pleural effusion was aspirated to obtain a tumour sample. She soon died of respiratory distress, but autopsy was not permitted.

### *Tissue preparation*

The surgically resected tumours were immediately frozen in OCT-compound (Tissue Tek Division, Miles Scientific Laboratories Inc, Napervill, Ill, USA) and stored at −80°C until use. Tumour were also fixed in 20% buffered formalin and embedded in paraffin for routine histological examination or fixed in 2.5% glutaraldehyde, dehydrated in graded alcohol and embedded in Epon 812 for electron microscopic observation. Cells in suspension were deposited on glass slides by centrifugation in a Cytospin (Shandon Southern Products Ltd, Cheshire, England) and were used for immunohistochemical study.

### *Immunohistochemistry*

Indirect immunoperoxidase staining was performed on acetone-fixed frozen sections by using monoclonal antibodies

(MoAbs). MoAb 5C11 was produced in our laboratory and its specificity was described previously (Hara et al., 1989). Briefly, 5C11 defines a cell surface protein with a molecular weight of 81,000 daltons preferentially expressed on ES but not on neuroblastoma and PNET cells. Two MoAbs, against neurofilament protein (NFP), anti-68 kilodalton (kD) NFP (Dakopatts, Glostrup, Denmark) and anti-200 kD NFP (Labsystem, Helsinki, Finland), were also used. In addition, anti-desmin (Dakopatts, Glostrup) was used in this study.

*Cell lines*

Four new ES cell lines were established in this study. Minced tumour tissues of Patients 1, 2 and 3, and cells obtained from the pleural effusion of Patient 4 were placed into culture dishes in RPMI 1640 medium supplemented with 10% foetal calf serum (RPMI/FCS). When cell growth became stable after several passages, cloning was carried out twice. Cells were treated with 0.25% trypsin, and 100 cells were suspended in 10 ml in medium and plated 10 cm dishes. Single cells were picked with a capillary pipette under reverse microscopy, and each was transferred to a well of a 96-well plate, and tumour cell lines were established. Chromosomes were analysed by trypsin G-banding as previously described (Homma et al., 1989). In addition to these new ES lines, four previously established ES lines were used. There were SCCH-196 (Homma et al., 1989), W-ES (Fujii et al., 1989), RD-ES, and SK-ES1. Both SCCH-196 and W-ES have been shown to have a chromosomal translocation with t(11;22)(q24;q12). RD-ES and SK-ES1 (Bloom, 1972) were obtained through the American Type Culture Collection (Rockville, Md, USA).

*Transplantation into nude mice*

Ten million tumour cells were transplanted into the backs of Balb/c nude mice according to the method previously described (Hata et al., 1980). When tumours reached to 10 mm in diameter, they were removed and processed for further characterisation.

In vitro *differentiation experiment*

Cells were plated on Lab-Tech 2-chamber slides or into 75 $cm^2$ culture flasks in RPMI/FCS at $1 \times 10^4 ml^{-1}$ with or without 2.5 mM $N_6$-$O_2$-dibutyryladenosine-3;5′-cyclic monophosphate (db-cAMP). db-cAMP was prepared by diluting a stock 125 mM solution dissolved in RPMI 1640. After 1 week of cultivation, the morphology of the cells on the chamber slides was observed, and cells were processed for immunohistochemistry and electron microscopy. Cells in the flasks were collected and were tested for indirect immunofluorescence with 5C11 followed by flow cytometrical analysis (Epics-Profile, Coulter Corp., Haileah, Fl, USA) as described previously (Hara et al., 1989).

### Results

*Original tumours*

Patients 1, 2 and 3 were treated by combined chemotherapy and/or radiotherapy before the tumours were removed. By light microscopy, they were found to be composed of small, round cells with clear cytoplasm and round to oval nuclei (Figure 1a). Most tumour cells of all three patients continued fine cytoplasmic granules which were positive in the periodic acid-Schiff reaction. They were digested with diastate, suggesting glycogen granules. In the tumours of patient 3, rosette-like structures were occasionally observed. Immunohistochemically, they all reacted with 5C11 (Figure 1b) but not with anti-68 kD NFP, anti-200 kD NFP, or anti-desmin. By electron microscopy, no evidence for neuronal differentiation such as neurosecretory granules or microtubules was observed. From these results the tumours in Cases 1, 2 and 3

**Figure 1** Histology of ES (Case 1, Original tumour of NCR-EW1), **a**, Hematoxylin eosin staining, showing small, round cells with round to oval nuclei and clear chromatin (original magnification × 100), **b**, 5C11 staining (original magnification × 100).

were diagnosed as ESs. In Case 4, only pleural effusion was obtained for analysis, but the tumour cells reacted with 5C11 and not with anti-200 kD NFP. Immunohistochemical findings as well as clinical manifestations favoured the diagnosis of ES in Case 4.

The tumours in Cases 3 and 4 were malignant small cell tumours of the thoracopulmonary region, but they had no evident phenotypical features of neurogenic tumours.

*Establishment of cell lines*

Four *in vitro* ES cell lines were newly established from tumours of seven patients with ES. All four lines, NCR-EW1, -EW2, -EW3, and -EW4 have been stably growing for more than 121, 78, 73 and 47 passages, respectively. NCR-EW1 grew with weak attachment to the bottom of the plastic flask and had round to spindle shaped cells. NCR-EW2 and -EW4 grew attached to the flask as well, with polygonal shaped cells, but occasionally they had cytoplasmic processes. In NCR-EW4, large flattened cells were occasionally seen. On the other hand, NCR-EW3 did not attach to the flask and grew as floating aggregates of cells in the medium. Cloning was carried out by the usual method and a few clones were obtained from each cell line. These cloned cells were shown to have cytological and immunocytological features similar to those of the mother cells morphologically and immunophenotypically. NCR-EW2, -EW3 and -EW4 reacted with 5C11, whereas NCR-EW1 did not. NCR-EW1 was derived from the transplanted tumour in Case 1 in nude mice which was used for the screening when 5C11 was produced. Through some unknown mechanism, 5C11 expression was lost during *in vitro* cultivation. NCR-EW1, -EW2, and -EW3 did not react with anti-68 kD or anti-200 kD NFP

MoAb. However, in NCR-EW4, some, but not all cells were found to be reactive with anti-200 kD NFP MoAb. When the staining profile was carefully examined, it was found that 200 kD NFP positive cells were located at the margin of the cell aggregates. As was stated above, the original tumour of NCR-EW4 (Case 4) was not positive for NFPs. Therefore, NCR-EW4 acquired the capability of differentiation towards the neuronal direction which becomes evident under certain culture conditions.

Doubling time was measured and chromosomal analysis was performed for NCR-EW1, -EW2, -EW3 and -EW4 at 104, 61, 56 and 30 passages, respectively and are summarised in Table I. In particular, a typical t(11;22)(q24;q12) chromosomal translocation was identified in NCR-EW2 (Figure 2). This abnormality was not observed in the other ES lines but an abnormality linked to chromosome No. 22 (+der(22)) was identified NCR-EW1 and -EW3. Of 15 metaphase cells from NCR-EW4, all had abnormal karyotypes but no specific abnormality could be detected. These results are shown in Table I.

The four new ES lines were examined for tumourigenicity in nude mice. Ten million tumour cells of each line were subcutaneously injected into five nude mice and all four lines produced tumours, in all the recipient mice. Histological examination revealed that the transplanted tumours were morphologically consistent with typical ES and no neuronal differentiation was observed by immunohistochemical and ultrastructural examination.

### In vitro *differentiation*

In addition to the four cell lines established in our laboratory, four other ES lines were used to examine their ability to differentiate in a neuronal direction. All these four cell lines, SCCH-196, W-ES, RD-ES and SK-ES1, reacted with 5C11. All lines except W-ES were negative for NFPs. W-ES was positive for both 68 kD and 200 kD NFPs although its original tumour was negative for these proteins (Fuji *et al.*, 1989).

When treated with db-cAMP, a morphological change was observed in NCR-EW2, RD-ES and SK-ES1. As is shown in Figure 3a, many cells of NCR-EW2 had elongated cytoplasmic processes after 3 days of treatment. A similar change was observed in RD-ES and SK-ES1. No apparent morphological

**Table I** Immunohistochemical reactivities and cytogenetical analyses of established cell line

| Cell lines | 5C11 | NFP 68 kD | NFP 200 kD | Des | D.T. | Chromosome | A/B |
|---|---|---|---|---|---|---|---|
| NCR-EW1 | – | – | – | – | 48 | 47,XY,-19,-22,+der(19)t(1;19)(q12;q11), +der(19)t(19;?)(p11;?),+der(22)t(?;22)(?;q11.2) | 4/6 |
| NCR-EW2 | + | – | – | – | 34.3 | 50,XY,+8,-13,+15,-17,+18,+18,+20,-21, del(3)(p21),t(11;22)(q24;q12), +2mark | 2/17 |
| NCR-EW3 | + | – | – | – | 55.4 | 41,del(X)(q26),-Y,-2,-4,-6,-9,-9,-10,-13,-14, -16,-17,-19,-22, del(3)(p12),del(8)(q22),del(11)(p12.2), +der(16)t(1;16)(q11;q11),+der(6)t(6;?)(p12;?),+der(9)t (9;?)(q34;?), +der(22)t(?;22)(?;q12), +4mark | 5/12 |
| NCR-EW4 | + | – | + | – | 40 | others | ?/15 |

NFP; neurofilament protein, Des; desmin, D.T.; doubling time (hour), A: Cells having chromosomal abnormalities, B: Examined cells at metaphase.

**Figure 2** Chromosomal analysis of NCR-EW2. Several pattern of chromosomal abnormalities were observed (arrows). NCR-EW2 has translocation t(11;22)(q24;q12) (arrow heads).

change was observed when cells were cultured in the absence of db-cAMP. Immunohistochemically, 200 kD NFP was detected in db-cAMP treated NCR-EW2, RD-ES and SK-ES1 as shown in Figure 3a. In particular, 200 kD NFP was usually detected in the elongated processes of tumour cells (Figure 3a), and 5C11 also reacted with them (Figure 3b). However, 68 kD NFP was not detected in db-cAMP treated cells. No NFP was detected immunohistochemically in the control culture. Electron microscopic observation of db-cAMP treated SK-ES1 (Figure 4a) revealed that microtubules in the cytoplasmic processes had clearly increased as compared with the control cell (Figure 4b) but neurosecretory granules were not found. When NCR-EW4 and W-ES were treated in the same manner, the number of 200 kD NFP positive cells increased. NCR-EW1, NCR-EW3 and SCCH-196, on the other hand, showed no morphological and immunohistochemical changes caused by the same treatment.

Expression of 5C11 by ES lines during *in vitro* treatment with db-cAMP was studied by flow cytometrical analysis. As is shown in Figure 5, the fluorescence intensity of 5C11 was weaker in the four cell ines (NCR-EW3, NCR-EWr, SCCH-196 and SK-ES1) than in the control culture. No definitive change in 5C11 expression was observed in NCR-EW2, RD-ES, and W-ES. These results are shown in Table II.

### Discussion

Differentiation diagnosis of ES and PNETs is often quite difficult because of their similar histological features (Triche & Cavazzana, 1987). Recently, they have been considered to be closely related because the same chromosomal abnormalities and the same proto-oncogene expression have been found in ES and PNET (Aurius et al., 1983; Turc-Carel et al., 1983; Whang-Peng et al., 1984; McKeon et al., 1988). We recently described the development of a new monoclonal antibody 5C11 which detects an 81,000 dalton protein cell surface expressed on ES cells (Hara et al., 1989). We have shown that ES can be clearly distinguished from PNET by 5C11 reactivity. Diagnostic criteria for PNET, as reported previously, include the ultrastructural and immunohistochemical findings of neurogenic tumours at initial diagnosis such as the ultrastuctural identification of neurosecretory granules and positive staining with anti-NFP antibodies. The evident relationship to neurogenic tissue further supports the diagnosis of PNET (Jaffe et al., 1984; Schmidt et al., 1985; Shinoda et al., 1988). ES, therefore, can be diagnosed as a tumour with no evidence of neuronal differentiation as stated above. By this criterion, 5C11 reacts with ES, but not with PNET. Such a strict diagnostic criterion is of particular importance when the biological characteristics of these tumours are studied.

The purpose of the present study was to determine the differentiation capability of ESs. For this purpose, eight ES cell lines including four newly established ones, NCR-EW1, -EW2, -EW3 and -EW4 established from extraosseous ESs were used. The pathologic characteristics of their original tumours satisfied the criteria referred to above (Navas-Palacios et al., 1984). Thus, the original tumours of all the new ES cell lines were 5C11 positive, but had no evident characteristics of neurogenic tumours. In all of the new lines except

**Figure 3** *In vitro* neuronal differentiation of ES lines. 200 kD NFP was usually detected in the elongated processes of NCR-EW2, **a**, and 5C11 also reacted with them, **b**, after db-cAMP treatment (original magnification × 100).

**Figure 4** Electron microscopic observation of SK-ES1 cells. **a**, db-cAMP treated SK-ES1, **b**, control cell (original magnification, **a**; × 1300, **b**; × 1,000).

NCR-EW1 5C11 expression has been stable for a long period of time. Reliability of 5C11 staining was also demonstrated in other ES cell lines already established. In contrast, cell lines established from PNET, NCR-PN1 (established in our laboratory from a sciatic nerve tumour in a 2 month old girl; manuscript in preparation) and PNET-Muraoka (provided by A. Nakagawara, Department of Pediatric Surgery, Kyushu University School of Medicine, Fukuoka, Japan; Hachitanda *et al.*, 1990) were negative for 5C11 (data not shown).

Some NCR-EW4 cells were found to express 200 kD NFP, although their original tumours were negative for the protein. Similarly, in W-ES, expression of 200 kD NFP was observed only in the cell line, but not in the original tumour (Fujii *et al.*, 1989). These results show that some ESs which do not have any characteristics of neurogenic tumours at their initial diagnosis can express neuronal markers in cell culture.

**Figure 5** Expression of 5C11 of ES cells was analysed by flow cytometer. **a**, **b**; SK-ES1, **c**, **d**; NCR-EW2. Cells were reacted with 5C11 (ii) not with class matched control antibody RI-10B5 (i) (Matsuura *et al.*, 1984). The x-axis shows the log green fluorescence intensity and y-axis represents the relative cell number. Not clearly decreased fluorescence intensity in SK-ES1 cells treated with db-cAMP, **b**, comparing with control culture, **a**, and unchanged intensity with, **d**, or without, **c**, db-cAMP treatment in NCR-EW2 cells.

**Table II** Characteristics of differentiation of Ewing's sarcoma

| *Group* | *Cell line* | *Immunostaining* *5C11* | *68 kD-NFP* | *200-NFP* | *Chromosome* |
|---|---|---|---|---|---|
| 1 | W-ES | + | + | + | t(11;22) |
| | NCR-EW4 | + (↓) | − | + | others |
| 2 | NCR-EW2 | | − (−) | − (+) | t(11;22) |
| | RD-ES | + | − (−) | − (+) | |
| | SK-ES1 | + | − (−) | − (+) | |
| 3 | NCR-EW3 | + (↓) | − (−) | − (−) | + der(22) |
| | SCCH-196 | + (↓) | − (−) | − (−) | t(11;22) |
| | NCR-EW1 | + (↓) | − (−) | − (−) | + der(22) |

Group 1: potential neuronal differentiation in an established cell line; 2: potential neuronal differentiation in an *in vitro* differentiation experiment; 3: no evidence of neuronal differentiation. +; positive, −; negative, ↓; reducing, ( ); after db-cAMP treatment.

Chromosomal analysis was performed on all new cell lines but the typical translocation involving t(11;22)(q24;q12) was seen only in NCR-EW2. However, a chromosomal abnormality, + der(22), was identified in NCR-EW1 and -EW3. Therefore it is likely that a structural change in chromosome 22 other than t(11;22)(q24;q12) is another characteristic feature of ESs. Precise characterisation of chromosomal abnormality will be fully described elsewhere (Kaneko *et al.*, manuscript in preparation).

Biological characteristics of ES were further determined by *in vitro* differentiation of eight ES cell lines stimulated with db-cAMP. Interestingly, a heterologous response to db-cAMP was observed. Three cell lines, NCR-EW2, RD-ES and SK-ES1 were shown to have morphological changes which were accompanied by the new expression of 200 kD NFP when stimulated with db-cAMP. In NCR-EW4 and W-ES, 200 kD NFP positive cells were clearly increase by db-cAMP treatment. On the other hand, NCR-EW1, -EW3 and SCCH-196 showed no definitive change after the same treatment in repeated experiments. It is of interest to compare our results with those of Cavazzana *et al.* (1987). By using five ES lines, they clearly demonstrated the capability of ES to differentiate in the neural direction after db-cAMP treatment. All the cell lines they used were derived from osseous ESs with a chromosomal abnormality involving t(11;12). Similarly, in our study, some ES lines could be induced to differentiate into 200 kD NFP positive neuronal cells. However, it must be emphasised that three cell lines could not be induced to differentiate by the same treatment. In addition, there was no correlation between differentiation capability and chromosomal abnormality. Therefore, it is likely that ES is a heterogeneous group with neuronal differentiation capability in at least three categories: (1) ES with potential neuronal differentiation which becomes evident in an established cell line, (2) ES with potential neuronal differentiation which becomes evident in an *in vitro* differentiation experiment and (3) ES with no evidence of neuronal differentiation even in a cell line (Table II). We propose this hypothesis in studying the biology of ES and its closely related tumour, PNET.

The relation between ES and PNET is still a problem to be solved. Recently, Marina *et al.* (1989) described putative diagnostic criteria for PNET. However, other authors show that some ESs can satisfy their criteria. For examples, some ESs show chromosomal abnormality t(11;22)(q24;q12) as shown here and in other reports and two PNET cell lines, NCR-PN1 and PNET-Muraoka, do not have such a translocation (unpublished observation; manuscript in preparation). Although they included neuron-specific enolase (NSE) and Leu 7 reactivity, we excluded these markers from our study. None of the antibodies available at present can specifically recognise the γγ-subunit of NSE, the form really specific to neuronal tissues, and we cannot rule out the possibility of a reaction with the αγ-subunit (Dranoff &

Bigner, 1984; Vinores *et al.*, 1984; Schmechel, 1985; Shimada *et al.*, 1988). When we stained malignant small round cell in childhood tumour (neuroblastomas, rhabdomyosarcomas, ESs and malignant lymphomas) with anti-NSE antiserum (Dakopatts), in fact, specific reactivity could not be obtained (data not shown). In addition, Leu 7 is not a specific marker for neuronal cells (Abo & Balch, 1981; Abo *et al.*, 1982). We therefore propose to use NFPs and 5C11 as immunohistochemical probes for differentiating between ES and PNET at the initial diagnosis.

It is of particular importance to determine as many characteristics (histology, immunohistochemistry, oncogene, chromosome, and establishment of cell lines) of these unique tumours as possible. Only through such extensive study the real features of ES and PNET can be obtained, and these features in turn should be utilised to established more effective therapeutic regimens. It is also important that investigators obtain a variety of probes to examine ES and PNET and in this regard we are ready to distribute monoclonal antibody 5C11 for more sophisticated study of this particular type of tumour.

We wish to thank Professor Osahiko Abe and Assistant Professor Jotaro Yokoyama, Department of Surgery, Keio University School of Medicine, for their scientific discussion, and Takashi Sekine, Division of Electron Microscopy, Tokai University School of Medicine, for his technical assistance. This work was supported by Grants-in-Aid for Cancer Research (3-6) from the Ministry of Health and Welfare and from the Ministry of Education of Japan, together with funds provided by the Entrustment of Research Program of the Foundation for Promotion of Cancer Research of Japan.

### References

ABO, T. & BALCH, C.M. (1981). A differentiation antigen of human NK and K cells identified by a monoclonal antibody (HNK-1). *J. Immunol.*, **127**, 1024.

ABO, T., COOPER, M.D. & BALCH, C.M. (1982). Postnatal expansion of natural killer and killer cell population in humans identified by the monoclonal HNK-1 antibody. *J. Exp. Med.*, **155**, 321.

ANGERVALL, L. & ENZINGER, F. (1975). Extraskeletal neoplasma resembling Ewing's sarcoma, *Cancer*, **36**, 240.

AURIUS, A., RIMBANT, C., BUFFE, D., DUBOSET, J. & MAZABRAUD, A. (1983). Chromosomal translocation in Ewing's sarcoma. *N. Engl. J. Med.*, **309**, 496.

BLOOM, E.T. (1972). Further definition by cytotoxicity tests of cell surface antigens of human sarcomas in culture. *Cancer Res.*, **32**, 906.

CAVAZZANA, A.O., MISER, J.C. & JEFFERSON, J. (1987). Experimental evidence for a neural origin of Ewing's sarcoma of bone. *Am. J. Pathol.*, **127**, 507.

DEHNER, L.P. (1986). Peripheral and central primitive neuroectodermal tumors. A nosological concept seeking a consensus. *Arch. Pathol. Lab. Med.*, **110**, 997.

DRANOFF, G. & BIGNER, D.D. (1984). A word of caution in the use of neuron-specific enolase expression in tumor diagnosis. *Arch. Pathol. Lab. Med.*, **108**, 535.

EWING, J. (1921). Diffuse endothelioma of bone. *Proc. N.Y Pathol. Soc.*, **21**, 17.

FUJII, Y., HONGO, T., NAKAGAWA, Y. & 5 others (1989). Cell culture of small round cell tumor originating in thoracopulmonary region: evidence for derivation from a primitive pluripotent cell. *Cancer*, **64**, 43.

HACHITANDA, Y., TSUNEYOSHI, M., ENJOJI, M., NAKAGAWARA, A. & IKEDA, K. (1990). Congenital primitive neuroectodermal tumor with epithelial and glial differentiation. *Arch. Pathol. Lab. Med.*, **114**, 101.

HARA, S., ISHII, E., TANAKA, S. & 4 others (1989). Monoclonal antibody specifically reactive with Ewing's sarcoma. *Br. J. Cancer*, **60**, 875.

HATA, J., UEYAMA, Y., TAMAOKI, N. & 5 others (1980). Human yolk sac tumor serially tranplanted in nude mice: its morphologic and functional properties. *Cancer*, **46**, 2446.

HOMMA, C., KANEKO, Y., SEKI, K., HARA, S., HATA, J. & SAKURAI, M. (1989). Establishment and characterization of a small round cell sarcoma cell line, SCCH-196, with t(11;22)(q24;q12). *Jpn. J. Cancer Res.*, **80**, 861.

JAFFE, R., AGOSTINI, B.S. Jr, SANTAMARIA, M. & 4 others (1984). the neuroectodermal tumor of bone. *Am. J. Surg. Pathol.*, **8**, 885.

JUERGENS, H., BIER, V., HARMS, D. & 8 others (1988). Malignant peripheral neuroectodermal tumors. A retrospective analysis of 42 patients. *Cancer*, **61**, 349.

MARINA, N.M., ETCUBANAS, E., PARHAMS, D.M., BOWMAN, L.C. & GREEN, A. (1989). Peripheral primitive neuroectodermal tumor (Peripheral neuroepithelioma) in children. A review of St Jude experience and controversies in diagnosis and management. *Cancer*, **64**, 1952.

MATSUURA, A., ISHII, Y., YUASA, H. & 4 others (1984). Rat T lymphocyte antigens comparable with mouse Lyt-1 and lyt-2, 3 antigenic systems: characterization by monoclonal antibodies. *J. Immunol.*, **132**, 316.

MCKEON, C., THIELE, C.T., ROSS, R.A. & 4 others (1988). Indistinguishable patterns of protooncogene expression in two distinct but closely related tumors: Ewing's sarcoma and neuroepithelioma. *Cancer Res.*, **48**, 4307.

NAVAS-PARACIOS, J.J., APARICIO-DUQUE, R. & VALDÉS, MD. (1984). On histogenesis of Ewing's sarcoma. An ultrastructural, immunohistochemical, and cytochemical study. *Cancer*, **53**, 1882.

SHIMADA, M., NEWTON, W.A, SOULE, E.H., QUALMAN, S.J., AOYAMA, C. & MAURER, H.M. (1988). Pathologic features of extraosseous Ewing's sarcoma: a report from the Intergroup Rhabdomyosarcoma Study. *Human Pathol.*, **19**, 442.

SCHMECHEL, D.E. (1985). γ-subunit of the glycolytic enzyme enolase: non-specific or neuron-specific? *Lab. Invest.*, **52**, 239.

SCHMIDT, D., HARMS, D. & BURDASH, S. (1985). Malignant peripheral neuroectodermal tumours of childhood and adolescence. *Virchows Arch. (Pathol. Anat.)*, **406**, 350.

SHINODA, M., TSUTSUMI, Y., HATA, J. & YOKOYAMA, S. (1988). Peripheral neuroepithelioma in childhood. *Arch. Pathol. Lab. Med.*, **112**, 1155.

TAFFT, M., VAUTER, G.F. & MITUS, A. (1969). Paravertebral small round cell tumors in children. *Radiology*, **92**, 1501.

TRICHE, T.J. & CAVAZZANA, A.O. (1987). Pathology of pediatric oncology. In Lipinscott, ?. (ed.), *Principles and Practice of Pediatric Oncology*, **93**.

TURC-CAREL, C., PHILIP, I., BERGER, M.P. & LENOIR, G.M. (1983). Chromosomal translocation in Ewing's sarcoma. *N. Engl. J. Med.*, **309**, 497.

VINORES, S.A., BONNIN, J.M., RUBINSTEIN, L.J. & MARANGOS, P.J. (1984). Immunohistochemical demonstration of neuron-specific enolase of the CNS and other tissues. *Arch. Pathol. Lab. Med.*, **108**, 536.

VOSS, B.L., PYSHER, T.J. & HUMPHREY, G.B. (1984). Peripheral neuroepitherioma in childhood. *Cancer*, **54**, 3059.

WHANG-PENG, J., TRICHE, T.J., KNUSTSEN, T., DOUGLAS, E.C. & ISRAEL, M.A. (1984). Chromosome translocation in peripheral neuroepithelioma. *N. Engl. J. Med.*, **311**, 584.